# Predictive factors of delayed bleeding after percutaneous nephrolithotomy requiring angioembolization

**DOI:** 10.1002/bco2.272

**Published:** 2023-07-24

**Authors:** Dariush Irani, Abdolreza Haghpanah, Alireza Rasekhi, Hooman Kamran, Mahdi Rahmanian, Mohammad Mehdi Hosseini, Behnam Dejman, Sajad Kiani

**Affiliations:** ^1^ Endourology Ward, Department of Urology Shiraz University of Medical Sciences Shiraz Iran; ^2^ Laparoscopy Research Center Shiraz University of Medical Sciences Shiraz Iran; ^3^ Department of Radiology Shiraz University of Medical Sciences Shiraz Iran; ^4^ Student Research Committee Shiraz University of Medical Sciences Shiraz Iran; ^5^ Medical School, MPH Department Shiraz University of Medical Sciences Shiraz Iran; ^6^ Department of Urology Shiraz University of Medical Sciences Shiraz Iran

**Keywords:** arteriovenous fistula, percutaneous nephrolithotomy, postoperative complications, postoperative haemorrhage, pseudoaneurysm

## Abstract

**Objectives:**

To investigate the predictive factors of delayed post‐percutaneous nephrolithotomy (PCNL) haemorrhage because of arteriovenous fistula (AVF) or pseudoaneurysm (PA) and compare the factors between AVF and PA.

**Patients and methods:**

This is a case–control study with a case‐to‐control ratio of 1:3. Out of 5077 patients who underwent PCNL from April 2015 to April 2018 in three different teaching hospitals, 113 had post‐PCNL haemorrhages because of AVF and/or PA. Seventy‐two patients met the inclusion criteria and entered the study as cases, while 216 patients without any postoperative complications were selected as controls.

**Results:**

Of all 72 studied patients with complications after PCNL, 35 (48.6%) had AVF, and the rest had PA. The regression model revealed that a history of diabetes (odds ratio [OR]: 2.799, 95% confidence interval [CI]: 1.392–5.630, *p*‐value = 0.004) and renal anomalies (OR: 2.929, 95% CI: 1.108–7.744, *p*‐value = 0.03) were associated with developing delayed post‐PCNL haemorrhage. However, no differences were seen between AVF and PA regarding selected variables (*p*‐value > 0.05).

**Conclusion:**

History of diabetes and renal anomalies were predictive factors for delayed post‐PCNL haemorrhage, but no predictive factors were found to differentiate PA and AVF from one another.

## 1 INTRODUCTION

Since its first introduction by Fernström et al. in 1976,^1^ percutaneous nephrolithotomy (PCNL) has gradually evolved and has become an effective therapeutic method for extracting large and complex renal calculi.^2^ However, it has a complication rate of about 20.5%–83%.^3^, ^4^

As one of the most frequent significant complications of PCNL, haemorrhage may be intraoperative or postoperative.^5^ Intraoperative haemorrhage is mainly due to larger tract size and multiple punctures.^6^, ^7^ On the other hand, postoperative bleeding can be early, which happens in the first 24 h after the operation, or delayed, within 1–3 weeks.^8^, ^9^ Early postoperative bleeding exists in the forms of bleeding from a nephrostomy tube, access tract bleeding and perinephric haemorrhage.^9^

Arteriovenous fistula (AVF) and pseudoaneurysm (PA) formation are the leading causes of delayed post‐PCNL haemorrhage, which need prompt attention.^9^ Severe post‐PCNL haemorrhage requiring intervention happens in less than 2% of patients, and in most cases, conservative treatment stops the bleeding.^10^, ^11^, ^12^ However, in patients with conservative management failure, angioembolization following angiography is the preferred option.^13^, ^14^, ^15^ Although post‐PCNL haemorrhage due to AVF or PA formation seems to be a rare phenomenon, it can be lethal if not diagnosed and treated well‐timed.^16^

This study aims to understand better the predictive factors of delayed post‐PCNL haemorrhage because of AVF or PA requiring angioembolization. Also, we hypothesized that these possible predictive factors might predict AVF and PA separately. Therefore, we compared the factors between the patients complicated with these two lesions to determine which can be a differentiating factor depending on the patient's and operative characteristics, as there were limited studies concerning this issue.

## 2 PATIENTS AND METHODS

### 2.1 Study design, setting and participants

In this case–control study, the medical records of 5077 patients who had undergone PCNL from April 2015 to April 2018 in Shahid Faghihi, Namazi and Aliasghar teaching hospitals were reviewed. These hospitals are run by the Shiraz University of Medical Sciences, Shiraz, Iran. In this period, 113 patients developed hematuria after the first 24 h of PCNL, which required angioembolization. Of these patients, 45, 64, and four had AVF, PA and both vascular lesions, respectively. Of these patients, 41 were omitted from the study because of incomplete medical records. For the control group, 216 patients who underwent PCNL and did not develop any complications were chosen through random sampling. In order to increase the statistical power of the study, we recruited multiple controls for each case at a 3:1 ratio (216:72).^17^

### 2.2 PCNL technique

Under general anaesthesia and prone position, procedures were done by two board‐certified endourologists with more than 5 years of experience. In all patients, a ureteral catheter was inserted before the operation. The bull's eye technique was used for fluoroscopy‐guided puncture and stone access.^18^ Of note, all procedures were done with infracostal punctures. The tract was dilated using a one‐step dilation technique until 30 Fr and then maintained by an Amplatz sheath. A pneumatic lithotripter and/or holmium laser were used to break down stones. A nephrostomy tube with/without a double J stent was used as needed at the end of the procedure. Stone removal was done in all studied patients through one access tract.

We performed all procedures in the study with just one infracostal percutaneous tract (to the chosen target calyx) using the bull's eye method, even in upper pole access and staghorn stones. However, if there was a significant residual stone after the operation, we used ancillary procedures such as shockwave lithotripsy (SWL), second‐time PCNL or retrograde intrarenal surgery (RIRS).

### 2.3 Angiography

After a diagnosis of iatrogenic hematuria, conservative treatment was performed. In the presence of persistent hematuria or haemoglobin drops, endovascular treatment was started, and angiography was the protocol of choice in emergency conditions. The treatment was a transarterial procedure. In the beginning, the right femoral artery was found by an interventional radiologist, and after puncture and passing the guidewire, an arterial sheath 5 Fr was inserted. A Cobra 5 Fr catheter was passed to the bleeding renal artery by a hydrophilic 0.035″ guidewire. After the injection of contrast (iopromide), the pathology was found. The feeder of the PA or AVF was super‐selectively catheterized by using a microcatheter and microwire. However, the treatments for AVF and PA are different. PA was embolized with a 1:1 mixture of cyanoacrylate glue and ethiodized oil. In this condition, the mixture was injected into the PA and its feeder by an experienced interventional radiologist. On the other hand, in AVF, the fistula was treated by a microcatheter and the insertion of microcoils. After these procedures, control angiography was performed, and after confirmation of the closure of pathologies, the procedure was terminated.

### 2.4 Data collection and variables

All data were collected by searching patients' medical records. We filled out a collection form for each of the cases and controls. Each form contained the patient's unit number, age, gender, history of diabetes, hypertension, SWL, PCNL on the same kidney, open surgery on the same kidney, renal anomaly, number of attempts to puncture, failed access, upper or mid‐pole access, stone location, side of the operated kidney, stone type, serum creatinine level before surgery, coagulation profile and size of the stone.

### 2.5 Data preparation

The preoperative serum creatinine level was categorized into less than 1.5 mg/dL or equal to or more than 1.5 mg/dL. International normalized ratio (INR) of more than 1.1, partial thromboplastin time (PTT) of more than 70 s or platelet count of less than 150 000/μL were considered coagulation profile abnormalities. The stone size was categorized into two groups: staghorn or non‐staghorn stones. The presence or absence of renal anomalies was evaluated according to previous radiologic studies, including computed tomography (CT) or intravenous urography (IVU).

### 2.6 Statistical analysis

Statistical Package for the Social Sciences (SPSS) software version 26 was used for analysis. We used frequency (n), percent of frequency (%), and mean ± standard deviation (SD) for descriptive analyses. The Pearson chi‐square test, Fisher's exact test and independent‐sample *t*‐test were used for bivariate analyses. A logistic regression model was applied to assess the independent association between developing AVF or PA after PCNL as the dependent variable and the considered factors. Variables with a *p*‐value of less than 0.1 in the bivariate analysis were selected to be included in the multivariable analysis. The adjusted odds ratio (OR) and its 95% confidence interval (CI) were estimated. A *p*‐value less than 0.05 was considered statistically significant.

### 2.7 Ethical approval

Informed consent was obtained from patients or their legal representatives. The Ethics Committee of Shiraz University of Medical Sciences reviewed and accepted the study protocol (approval code: IR.SUMS.MED.REC.1397.505).

## 3 RESULTS

Of all 72 studied patients with delayed post‐PCNL haemorrhage requiring angioembolization, 35 (48.6%) developed AVF, and the rest had PA. Besides, the success rate of angioembolization was 100% in all cases. There was no statistical difference between the mean age of cases (48.17 ± 11.37 years) and controls (46.73 ± 13.70 years) (*p*‐value = 0.426). The female/male ratios were 1.57 (44:28) and 1.05 (111:105) among cases and controls, respectively, and there was no statistical difference between the gender distribution of cases and controls (*p*‐value = 0.152). Also, the mean time to onset of haemorrhage after PCNL was 17.16 days (range: 3–40 days).

Table 1 shows the demographic and PCNL‐related factors of cases and controls. Bivariate analysis showed a statistically significant difference between cases and controls regarding a history of diabetes, hypertension, SWL, PCNL, open surgery, coagulation disorder, staghorn stone and renal anomalies. However, failed access, multiple attempts to puncture, access pole, stone location, side of the operation, stone type and preoperative serum creatinine were not significantly different between cases and controls.

**TABLE 1:** Demographic and PCNL‐related factors of cases and controls.

Factors	Patients		*p*‐value*
	Cases; *n* (%)	Controls; *n* (%)	
History of diabetes	30 (41.7)	32 (14.8)	<0.001
History of hypertension	43 (59.7)	71 (32.9)	<0.001
History of SWL	47 (65.3)	95 (44.0)	0.002
History of PCNL	47 (65.3)	99 (45.8)	0.004
History of open surgery	20 (27.8)	36 (16.7)	0.039
Coagulation disorder	9 (12.5)	9 (4.2)	0.011
Staghorn stone	25 (34.7)	45 (20.8)	0.017
Renal anomaly	17 (23.6)	12 (5.6)	<0.001
Failed access	7 (9.7)	15 (6.9)	0.442
Multiple attempts to puncture	5 (6.9)	11 (5.1)	0.552
Upper or mid pole access	11 (15.3)	23 (10.6)	0.292
Stone location			
Upper pole	2 (2.8)	9 (4.2)	0.128
Mid pole	6 (8.3)	32 (14.8)	
Lower pole	21 (29.2)	61 (28.2)	
Renal pelvis	18 (25.0)	69 (31.9)	
Mid pole, lower pole and renal pelvic (staghorn)	25 (34.7)	45 (20.8)	
Side of operated kidney			
Right	30 (41.7)	98 (45.4)	0.584
Left	42 (58.3)	118 (54.6)	
Stone type			
Calcium oxalate and calcium phosphate	54 (75.0)	174 (80.6)	0.693
Struvite	11 (15.3)	29 (13.4)	
Uric acid	4 (5.6)	8 (3.7)	
Cystine	3 (4.2)	5 (2.3)	
Preoperative serum creatinine			
<1.5 mg/dL	54 (75.0)	176 (81.5)	0.235
≥1.5 mg/dL	18 (25.0)	40 (18.5)	

Abbreviations: PCNL, percutaneous nephrolithotomy; SWL, shockwave lithotripsy.

* Pearson chi‐square test.

Of note, among the 17 (23.6%) patients with a renal anomaly in the study group, 12 (70.6%) had a horseshoe kidney and five (29.4%) had a malrotated kidney. Besides, in the control group, 12 patients (5.6%) had a renal anomaly, of which 10 (83.3%) had a horseshoe kidney and two (16.7%) had a malrotated kidney. Although the presence of renal anomalies was different between cases and controls (*p*‐value < 0.001), the type of anomaly (horseshoe kidney or malrotated kidney) was not different (*p*‐value = 0.665) (Table 2).

**TABLE 2:** Renal anomaly in cases and controls.

Renal anomaly	All; *n* (%)	Cases; *n* (%)	Controls; *n* (%)	*p*‐value^*^
Horseshoe kidney	22 (75.9%)	12 (70.6%)	10 (83.3%)	0.665
Malrotated kidney	7 (24.1%)	5 (29.4%)	2 (16.7%)	

* Fisher's exact test.

Table 3 shows the demographic and PCNL‐related factors of cases based on either the complication of AVF or PA. The mean age was 46.50 ± 7.26 and 49.70 ± 14.07 years in the AVF and PA groups, respectively (*p*‐value = 0.228). The gender distribution was not significantly different between the groups (female/male: 24:11 in AVF and 20:17 in PA; *p*‐value = 0.207). Besides, none of the selected factors had a statistically significant difference between the two groups of AVF and PA.

**TABLE 3:** Demographic and PCNL‐related factors of cases based on either complication.

Factors	Type of complications		*p*‐value*
	AVF; *n* = 35	PA; *n* = 37	
Age, year; mean ± SD	46.50 ± 7.26	49.70 ± 14.07	0.228
Gender; *n* (%)			
Female	24 (68.6)	20 (54.1)	0.207
Male	11 (31.4)	17 (45.9)	
History of diabetes; *n* (%)	13 (37.1)	17 (45.9)	0.449
History of hypertension; *n* (%)	19 (54.3)	24 (64.9)	0.360
History of SWL; *n* (%)	24 (68.6)	23 (62.2)	0.568
History of PCNL; *n* (%)	24 (68.6)	23 (62.2)	0.568
History of open surgery; *n* (%)	7 (20.0)	13 (35.1)	0.152
Coagulation disorder; *n* (%)	2 (5.7)	7 (18.9)	0.153
Staghorn stone; *n* (%)	11 (31.4)	14 (37.8)	0.568
Renal anomaly; *n* (%)	7 (20)	10 (27.0)	0.483
Failed access; *n* (%)	2 (5.7)	5 (13.5)	0.430
Multiple attempts to puncture; *n* (%)	2 (5.7)	3 (8.1)	1.000
Upper or mid pole access; *n* (%)	5 (14.3)	6 (16.2)	0.820
Stone location; *n* (%)			
Upper pole	1 (2.9)	1 (2.7)	0.871
Mid pole	4 (11.4)	2 (5.4)	
Lower pole	11 (31.4)	10 (27.0)	
Renal pelvis	8 (22.8)	10 (27.0)	
Mid pole, lower pole and renal pelvic (staghorn)	11 (31.4)	14 (37.8)	
Side of operated kidney; *n* (%)			
Right	13 (37.1)	17 (45.9)	0.449
Left	22 (62.9)	20 (54.1)	
Stone type; *n* (%)			
Calcium oxalate and calcium phosphate	25 (71.4)	29 (78.3)	0.644
Struvite	5 (14.3)	6 (16.2)	
Uric acid	3 (8.6)	1 (2.7)	
Cystine	2 (5.7)	1 (2.7)	
Preoperative serum creatinine; *n* (%)			
<1.5 mg/dL	25 (71.4)	29 (78.3)	0.496
≥1.5 mg/dL	10 (28.6)	8 (21.7)	

Abbreviations: AVF, arteriovenous fistula, PA, pseudoaneurysm; PCNL, percutaneous nephrolithotomy; SD, standard deviation; SWL, shockwave lithotripsy.

* Independent sample *t*‐test or Pearson chi‐square test/Fisher's exact test.

A logistic regression model was performed to ascertain the effects of a history of diabetes, hypertension, SWL, PCNL, open surgery, coagulation disorder, staghorn stone and renal anomalies on the likelihood of developing delayed post‐PCNL haemorrhage because of AVF or PA after PCNL. Multivariable analysis revealed that only a history of diabetes (OR: 2.799, 95% CI: 1.392–5.630, *p*‐value = 0.004) and renal anomalies (OR: 2.929, 95% CI: 1.108–7.744, *p*‐value = 0.03) were associated with developing delayed post‐PCNL haemorrhage (Table 4).

**TABLE 4:** Results of binary logistic regression.

Parameters	Regression coefficient	Standard error	*p*‐value	Odds ratio	95% CI of OR
Intercept	−2.122	0.6795	0.002	0.12	0.032–0.454
History of diabetes*	1.029	0.3565	0.004	2.799	1.392–5.630
History of hypertension*	0.332	0.3422	0.332	1.394	0.713–2.725
History of SWL*	0.451	0.3172	0.155	1.569	0.843–2.922
History of PCNL*	0.511	0.3239	0.115	1.666	0.883–3.144
History of open surgery*	0.007	0.4015	0.986	1.007	0.458–2.212
Coagulation disorder*	0.148	0.6037	0.807	1.159	0.355–3.785
Staghorn/non‐staghorn stone**	0.067	0.3656	0.854	1.070	0.522–2.190
Renal anomaly*	1.075	0.4960	0.03	2.929	1.108–7.744

*Note*: Developing AVF or PA as the dependent variable.

Abbreviations: CI, confidence interval; OR, odds ratio; PCNL, percutaneous nephrolithotomy; SWL, shockwave lithotripsy.

* Negative state as reference.

** Non‐staghorn stone as reference.

## 4 DISCUSSION

Delayed post‐PCNL haemorrhage, usually presenting with intermittent hematuria, is a rare phenomenon mainly because of AVF or PA formation and can generally be controlled by angiography with embolization. In the case of dealing with delayed post‐PCNL haemorrhage, angiography is the gold standard procedure, which provides us with diagnosing and treating a vascular lesion in one session. If angiography fails, open vascular repair, or at last, nephrectomy, may be used to control the haemorrhage.^8^, ^19^, ^20^, ^21^ This study found that a history of diabetes and renal anomalies was associated with higher odds of developing delayed post‐PCNL haemorrhage because of AVF or PA requiring angioembolization. However, there were no significant differences between AVF and PA regarding the recorded factors.

Excluding the patients with both lesions, the ratio of PA:AVF was 1.42 in our study. The predominance of PA was similarly shown in the studies by Srivastava et al. (PA:AVF = 2.16),^5^ El‐Nahas et al. (PA:AVF = 2.22)^22^ and Jinga et al. (PA:AVF = 3.00).^23^ However, a study by Un et al. (PA:AVF = 0.20)^16^ showed an inverse ratio. We have no justification to explain the reason for the predominance of PA compared with AVF.

As PA is formed because of damage to the renal arterial lumen, AVF is developed by combined arterial and venous damage, and more importantly, the radiological treatment procedures of these two lesions are different (as previously explained), so we compared the variables between AVF and PA. However, there were no differences between AVF and PA concerning the variables in our study. This shows that none of the variables can predict the underlying cause of post‐PCNL bleeding between PA and AVF. To the best of our knowledge, previous studies have not compared the possible predictor factors between these two lesions. We hypothesized that the predisposing factors for PA and AVF might be different, but the study results did not confirm our hypothesis. We suggest further studies to compare these two causes of post‐PCNL bleeding.

This study found a history of diabetes as a risk factor for developing delayed post‐PCNL haemorrhage. According to another study done by Akman et al.,^24^ diabetes was a predictive factor for bleeding during PCNL. However, some other studies did not confirm this result for post‐PCNL haemorrhage.^25^, ^26^ In a study done by Hart and Cohen,^27^ it was reported that capillary fragility is increased in the setting of diabetes. And as a result, it may increase the chance of vascular injury and delayed post‐PCNL haemorrhage. Also, the correlation between diabetes and atherosclerosis has been speculated as one of the reasons.^28^ Therefore, optimizing haemoglobin A1c (Hb1Ac) before elective kidney stone surgery is recommended and may prevent post‐PCNL haemorrhage, but further studies have to be performed to evaluate this issue.

We also found that renal anomaly is a risk factor for post‐PCNL severe haemorrhage. In concordance with the study results performed by Un et al.,^16^ a renal anomaly may increase the risk of vascular injury and, as a result, the chance of post‐PCNL severe haemorrhage. Also, in a study by Binbay et al.,^29^ the haemoglobin decrease after PCNL in patients with malrotated kidneys was higher than in the controls. In contrast, PCNL of the horseshoe kidney has been reported to be safe,^30^ and many studies have reported that renal anomaly is not a risk factor for post‐PCNL bleeding.^22^, ^31^ Hence, further and more comprehensive studies are needed to evaluate post‐PCNL haemorrhage in renal anomalies.

Srivastava et al.^5^ studied 1854 patients for post‐PCNL haemorrhage; 27 underwent angioembolization because of severe bleeding. Thirteen patients had PA, six had AVF, four had a combination of both, one had a lumbar artery injury, and three had no lesion. Of all factors, including stone size, punctures, simultaneous bilateral procedures, intraoperative pelvic perforation, chronic renal failure and blood transfusion, only stone size was the predicting factor for post‐PCNL haemorrhage. In our study, however, although staghorn stones were associated with more chance of developing delayed post‐PCNL haemorrhage, according to the bivariate analysis, the result was not confirmed by the multivariable analysis. Most of the discussed studies agreed with increased odds of post‐PCNL severe haemorrhage and, as a result, angiography requirements with increased stone size and complexity,^5^, ^16^, ^22^, ^23^, ^25^, ^26^ contrary to our research. In addition, in a study by Jinga et al.,^23^ by evaluating the severity of hematuria after PCNL, the authors showed that even the severity is influenced by the mean stone size. Of note, larger and more complex stones may require more access tracts to be obliterated, and previous studies have stated multiple access tracts as a predictive factor for post‐PCNL severe bleeding.^22^, ^25^ So, it is possible that stone size and complexity indirectly affect post‐PCNL severe haemorrhage through the number of access tracts. Therefore, one of the main reasons for the non‐significant result of stone size in our study may be that none of the cases had more than one access tract.

Although in many previous studies, staghorn stones were associated with more bleeding,^22^, ^23^ a study by Dong et al.^32^ reported different results; in the mentioned study, no staghorn calculi were shown to be a risk factor for severe bleeding. The authors stated that better kidney tolerance in patients who have staghorn stones without symptoms may be one of the reasons for that conclusion.

Some previous studies have studied a number of additional probable risk factors. Nouralizadeh et al.^33^ reported the tubeless PCNL as a predictive factor for delayed post‐PCNL haemorrhage. However, we do not perform tubeless PCNL in our centres, and in all cases in this study, a nephrostomy tube was inserted. Besides, in a study by Kim et al.,^34^ puncture correctness evaluated by the postoperative CT scan was the only predictor of severe bleeding requiring angioembolization. Although we assessed failed access and upper or mid‐pole access, showing no significant association with post‐PCNL bleeding, a postoperative CT scan was not performed in our study as it is not routine postoperative management. Therefore, we recommend future studies to include additional variables, especially to compare the predictive factors of PA with AVF, as this comparison is lacking in the literature.

### 4.1 Limitations

This study was not without limitations. The sample size was relatively small. However, it was inevitable considering the low prevalence of post‐PCNL bleeding. Besides, our study was a retrospective study, which may cause data gaps and missing data to present some variables, including a stone nephrolithometry scoring, and has its limitations compared with prospective research. The study was retrospective due to the low prevalence of post‐PCNL bleeding. Also, as we performed all of the procedures with one infracostal puncture, the role of multi‐tract PCNL in post‐PCNL bleeding could not be determined.

## 5 CONCLUSIONS

In conclusion, AVF and PA are the two leading causes of delayed post‐PCNL haemorrhage that happen in a few cases. We concluded that a history of diabetes and having a renal anomaly were predictive factors for delayed post‐PCNL haemorrhage. Therefore, optimizing Hb1Ac before elective kidney stone surgery is recommended. However, no variable was found to be a differentiating predictor of PA and AVF.

## AUTHOR CONTRIBUTIONS

Dariush Irani and Abdolreza Haghpanah designed the study. Behnam Dejman and Sajad Kiani performed the acquisition of data. Mahdi Rahmanian analysed and interpreted the data. Behnam Dejman and Hooman Kamran drafted the manuscript. Hooman Kamran, Abdolreza Haghpanah, Alireza Rasekhi, Dariush Irani and Mohammad Mehdi Hosseini revised the manuscript. All authors read and approved the final manuscript.

## CONFLICT OF INTEREST STATEMENT

Not applicable.
